# Prevalence of latent tuberculosis infection among coal workers’ pneumoconiosis patients in China: a cross-sectional study

**DOI:** 10.1186/s12889-018-5373-1

**Published:** 2018-04-11

**Authors:** Yan Jin, Huanqiang Wang, Jianfang Zhang, Chunguang Ding, Ke Wen, Jingguang Fan, Tao Li

**Affiliations:** 1 National Institute of Occupational Health and Poison Control, Chinese Center for Disease Control and Prevention, Beijing, China; 2 Department of Infectious Disease, Taizhou Center for Disease Control and Prevention, Taizhou, China; 3 Research Center for Occupational Safety and Health, State Administration of Work Safety, Beijing, China

**Keywords:** *Mycobacterium tuberculosis*, IFN-γ, Occupational health

## Abstract

**Background:**

Little is known about the prevalence of latent tuberculosis infection (LTBI) among coal workers’ pneumoconiosis (CWP) patients. To estimate the prevalence of LTBI and identify its associated risk factors among CWP patients.

**Methods:**

A cross-sectional study was conducted to assess the prevalence of LTBI. Participants were screened for active TB or a history of TB by X-ray and those that underwent QuantiFERON-TB Gold In-Tube (QFT) test. A standardized questionnaire was completed and risk factors were assessed for acquiring TB. Log-binomial regression was used to estimate the LTBI prevalence ratio (PR) in relation to risk factors.

**Results:**

Of 244 individuals with CWP (median age 67 years; all male), 162 (66.4%) were QFT positive. In Multivariate analysis, poor workplace ventilation (adjusted prevalence ratio [APR] = 1.26) and intake of fruits regularly (≥4 days of every week) (APR = 0.81) (all ***p*** < 0.05) were associated with a decreased risk of QFT.

**Conclusions:**

This study showed a high prevalence of LTBI among individuals with CWP in China. Poor workplace ventilation may be an important contributing factor for LTBI. Regular monitoring and dust control measures need to be improved in workplaces to ensure the safety of workers. Moreover, intake of fruits regularly may be a protective factor for LTBI. However, the effect of fruits should be further studied.

**Electronic supplementary material:**

The online version of this article (10.1186/s12889-018-5373-1) contains supplementary material, which is available to authorized users.

## Background

China currently has one of the highest rates of pneumoconiosis in the world, with this disease accounting for over 85% of all reported occupational diseases in the country [1, 2]. Coal workers’ pneumoconiosis (CWP) is believed to be the predominant type, accounting for about 60% of the total number of new cases of pneumoconiosis [1, 2]. Tuberculosis (TB) is the most common complication associated with CWP [3]. However, little is known about the prevalence of latent TB infections (LTBI) among CWP patients. A LTBI is defined as the presence of immune responses to *Mycobacterium tuberculosis* antigens without any clinical evidence of active TB. Patients with LTBI are at risk of developing active TB disease and becoming infectious [4]. The risk of LTBI reactivation can be reduced by preventive treatment. Identification and treatment of LTBI in individuals at high risk of developing active disease has been practiced as an effective strategy for TB control in the US [5].

The World Health Organization has issued guidelines on the management of LTBI for a wide range of risk groups, including patients with HIV or silicosis [6]. There is no diagnostic gold standard test for LTBI, but *M. tuberculosis*-specific interferon (IFN)-γ-based diagnostic tests offer increased specificity (93%–99%) and at least equivalent sensitivity (75%–90%) as the tuberculin skin test (TST) and are unaffected by previous BCG vaccination [7]. In Norway, a limited number of studies on the results of IFN-γ release assays (IGRAs) in various populations have been published [8–10], but no study has focused on CWP. Only one study has reported the prevalence of LTBI among aged underground hard coal miners using IGRAs in Germany [11]. The aim of the current study was to estimate the prevalence of LTBI using a T-cell-based IGRA in CWP patients and to determine the risk factors associated with a positive test result.

## Methods

### Study participants

This cross-sectional study was conducted from October to December, 2016, as part of a pneumoconiosis screening program at the Hospital of Occupational Diseases in Beijing. This hospital has seven wards specialized in pneumoconiosis, with a total capacity of 380 beds. Study participants were recruited from all the wards within the hospital. A total of 376 participants diagnosed CWP based on the China National Diagnostic Criteria for Pneumoconiosis by performing a full chest X-ray of good quality. Diagnoses were made independently by three certified doctors on the basis of occupational history, physical examination, chest radiograph and pulmonary function tests. All participants were acquired that had no history of TB and received a medical evaluation and chest X-rays to exclude active TB.

### IGRA test

Because there is no gold standard for the diagnosis of LTBI, we chose a T-cell-based IGRA for this study which was performed according to the manufacturer’s instructions (QuantiFERON-TB Gold InTube, Cellestis Limited, Carnegie, Australia). A venous blood sample (1 ml) was collected from each individual and aliquoted into three tubes (one containing TB-specific antigens, one containing mitogen and a negative control tube). The samples were transported within 4–6 h of collection and incubated for 24 h at 37 °C. Then the samples were centrifuged at 3000 × rcf for 10 min, and the plasma was collected and stored at 4 °C until the IGRA was performed using the enzyme-linked immunosorbent assay (ELISA) kit provided with the TB-Gold tube. The optical density (OD) of each sample was determined using a 450-nm filter and a 620-nm reference filter on an ELISA plate reader. The results were interpreted as positive, negative or indeterminate on the basis of the manufacturer’s recommended cut-off values (IFN-γ ≥ 0.35 IU/ml) using QuantiFERON-TB Gold In Tube (QFT) analysis software developed by the company.

### Questionnaire

Information on the following variables was collected using a standardized questionnaire. Data obtained included, among others, age, BMI (body mass index), highest educational level, marital status and personal income. We also inquired about occupational factors including stages of CWP, job category, years of work, dust exposure period, age at first dust exposure and duration of dust exposure. We also assessed environmental and behavioral factors including, among others, workplace ventilation, accommodation, smoking and alcohol consumption. We also assessed family history of TB, close contact with patients with TB and BCG vaccination.

### Statistics

QFT laboratory results and questionnaire data were entered into the EpiData software v3.1 (EpiData Association, Odense, Denmark) and were analyzed using SAS software (Version 9.4, SAS Institute, USA). Continuous variables were described by medians and interquartile range (IQR). Proportions were summarized for categorical variables. The prevalence of LTBI was estimated by dividing the number of participants with a positive QFT test result by the total number of participants. Log-binomial regression with a logarithmic link function was used to estimate prevalence ratios (PRs) with their 95% confidence intervals; this was preferred to logistic regression as odds ratios tend to over-state effect sizes, particularly when prevalence is greater than 10% [12]. Bivariate analyses of each potential risk factor and LTBI were done first. Variables with *p* values< 0.05 in bivariate analyses were included in multivariate log-binomial regression analyses. The significance level for testing associations was set at 0.05. All statistical tests were two tailed.

## Results

**Fig. 1:**
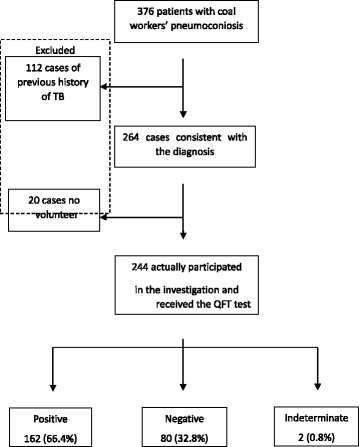
Study participants flow chart. Flow diagram for 244 patients with coal workers’ pneumoconiosis who participated in the investigation and received the QFT test

In total, 376 patients admitted to the Hospital of Occupational Diseases and screened for pneumoconiosis from October to December 2016, were selected for this survey. Of these, 112 (29.8%) were excluded owing to a previous history of TB. Of the remaining 264, 20 (5.3%) did not volunteer to provide blood samples. Therefore, 244 (64.9%) participants actually participated in the investigation and received the QFT test (Fig. 1). The participants were all male and ranged from 42 to 92 years, with a median age of 67 years. The BMI distribution showed that more than half of the participants (59.3%) were overweight. About half of the participants (50.4%) were of primary school or lower education level. Personal income per month showed that more than 70% of the participants earned between 3000 and 4000 RMB, and family income per head per month showed that 67.2% of the participants earned below 6000 RMB. About 90% of the participants were born in the countryside (Table 1).

**Table 1:** Characteristics of study participants received the QFT test in the Hospital of Occupational Diseases (*n* = 244)

Characteristics	n(%)or median(IQR)
Sex	
Male	244(100.0)
Female	0(0.0)
Age(years)	67(64–75)
< 60	20(8.2)
60~ 70	125(51.2)
≥ 70	99(40.6)
Ethnic origin	
Ethnic Han	242(99.2)
Other ethnic groups	2(0.8)
BMI(kg/m2) ^a^	25.6(23.5–27.3)
18.5~ 25	99(40.7)
≥ 25	144(59.3)
Highest education level	
Primary school or lower	123(50.4)
Middle school	95(38.9)
High school	26(10.7)
Marital status	
Married	229(93.9)
Divorced or Widowed	15(6.1)
Personal income per month (RMB)	3050(3000–3700)
< 3000	29(11.9)
3000~ 4000	178(72.9)
≥ 4000	37(15.2)
Family income per head per month(RMB)^a^	4500(3500–6000)
< 6000	137(67.2)
6000~ 10,000	49(24.0)
≥ 10,000	18(8.8)
Place of Birth	
City	23(9.4)
Countryside	221(90.6)

*QFT* QuantiFERON-TB Gold In-Tube, *IQR* interquartile range, *BMI* body mass index, *RMB* Renminbi

^a^ Sum may not equal the total because of missing data

The prevalence of LTBI in the 244 CWP patients, based on QFT positivity, was 66.4% (95% CI: 61.0%–72.9%) (Additional file 1). The QFT response was negative in 32.8% of cases and was indeterminate in 0.8% (Fig. 1). Excluding two cases for which the QFT results were indeterminate, the remaining 242 CWP cases were analyzed for risk factors. As shown in Table 2, there were no significant difference in the prevalence of LTBI in terms of socio-demographic characteristics and occupational factors. The environmental risk factor for QFT was workplace ventilation. Using the subgroup workplace ventilation as the reference group, the prevalence ratio [PR] was elevated for poor workplace ventilation (PR: 1.25; 95% confidence interval [CI]: 1.04–1.51). The behavioral protective factor for QFT was intake of fruits regularly. Using the subgroup < 1 day of every week of intake of fruit as the reference group, the risk of LTBI was reduced by 21% both for those eating fruit 1 to 3 days of every week (PR: 0.79; 95% CI: 0.63–0.99) and those eating fruit ≥4 days of every week (PR: 0.79; 95% CI: 0.64–0.97).

**Table 2:** Bivariate analyses of factors associated with QFT positivity among study participants (*n* = 242)

Factors	QFT test		Prevalence ratio (95% CI)	*p*-value
	Negative	Positive		
	80(33.1%)	162(66.9%)		
Socio-demographic characteristics				
Age (years)				
< 60	9(45.0%)	11(55.0%)	1	–
60-	43(34.4%)	82(65.6%)	1.19(0.79–1.81)	0.407
≥ 70	28(28.9%)	69(71.1%)	1.29(0.85–1.96)	0.226
BMI (kg/m^2^) ^a^				
18.5-	35(36.1%)	62(63.9%)	1	–
≥ 25	44(30.6%)	100(69.4%)	1.09(0.90–1.31)	0.379
Highest education level				
Primary school or lower	43(35.5%)	78(64.5%)	1	–
Middle school	28(29.5%)	67(70.5%)	1.09(0.91–1.32)	0.342
High school	9(39.1%)	17(73.9%)	1.01(0.74–1.38)	0.928
Marital status				
Married	73(32.0%)	155(68.0%)	1	–
Divorced or Widowed	7(50.0%)	7(50.0%)	0.73(0.43–1.25)	0.257
Personal income per month (RMB)				
< 3000	7(24.1%)	22(75.9%)	1	–
3000-	60(34.1%)	116(65.9%)	0.87(0.69–1.09)	0.233
≥ 4000	13(35.1%)	24(64.9%)	0.86(0.62–1.17)	0.323
Family income per head per month (RMB) ^a^				
< 6000	45(33.3%)	90(66.7%)	1	–
6000-	16(32.7%)	33(67.3%)	1.01(0.80–1.27)	0.931
≥ 10,000	5(27.8%)	13(72.2%)	1.08(0.79–1.48)	0.613
Place of birth				
City	7(30.4%)	16(69.6%)	1	–
Countryside	73(33.3%)	146(66.7%)	0.96(0.72–1.28)	0.771
Occupational factors				
Stages of CWP				
I	62(31.3%)	136(68.7%)	1.00	–
II	16(39.0%)	25(61.0%)	0.89(0.68–1.15)	0.374
III	2(66.7%)	1(33.3%)	0.48(0.10–2.41)	0.377
Job category				
Transport and helping	9(60.0%)	6(40.0%)	1.00	–
Mining	31(36.5%)	54(63.5%)	1.59(0.84–3.01)	0.157
Tunneling	16(25.4%)	47(74.6%)	1.87(0.10–3.52)	0.055
Both tunneling and mining	24(30.4%)	55(69.6%)	1.74(0.92–3.29)	0.088
Years of work				
10-	4(40%)	6(60.0%)	1.00	–
20-	21(27.6%)	55(72.4%)	1.21(0.71–2.04)	0.484
30-	47(33.3%)	94(66.7%)	1.11(0.66–1.87)	0.691
40-	8(53.3%)	7(46.7%)	0.78(0.37–1.63)	0.506
Dust exposure period				
1940-	13(40.6%)	19(59.4%)	1.00	–
1960-	47(29.9%)	110(70.1%)	1.18(0.87–1.60)	0.286
1980-	20(37.7%)	33(62.3%)	1.05(0.74–1.50)	0.793
Age at first dust exposure				
< 18	9(33.3%)	18(66.7%)	1.00	–
18–29	63(32.8%)	129(67.2%)	1.01(0.76~ 1.34)	0.286
≥ 30	8(34.8%)	15(65.2%)	0.98(0.66~ 1.46)	0.793
Duration of dust exposure(years)				
< 10	1(14.3%)	6(85.7%)	1.00	–
10-	21(38.9%)	33(61.1%)	0.71(0.49–1.03)	0.073
20-	33(34.4%)	63(65.6%)	0.77(0.55~ 1.07)	0.119
30-	25(29.4%)	60(70.6%)	0.82(0.59–1.15)	0.252
Environmental factors				
Workplace ventilation				
Well	46(41.1%)	66(58.9%)	1.00	–
Poor	34(26.2%)	96(73.8%)	1.25(1.04–1.51)	0.017
Accommodation				
Building	36(32.1%)	76(67.9%)	1.00	–
Bungalow	44(33.8%)	86(66.2%)	0.98(0.82–1.16)	0.779
Living space^b^				
< 20	21(29.2%)	51(70.8%)	1.00	–
20–40	35(34.0%)	68(66.0%)	0.93(0.761–1.142)	0.497
40-	6(23.1%)	20(76.9%)	1.10(0.873–1.381)	0.424
Behavioral factors				
Smoking				
Never	12(34.3%)	23(65.7%)	1.00	–
Once	45(38.5%)	72(61.5%)	0.94(0.71–1.24)	0.497
Occasionally	4(66.7%)	2(33.3%)	0.51(0.16–1.61)	0.424
Frequently	19(22.6%)	65(77.4%)	1.18(0.90–1.54)	0.424
Start smoking age				
< 18	14(31.8%)	30(68.2%)	1.00	–
≥ 18	51(32.3%)	107(67.7%)	0.99(0.79–1.25)	0.954
Smoking index^c^				
0	12(34.3)	23(65.7)	1	–
> 0	21(36.2)	37(63.8)	0.99(0.73–1.35)	0.954
15-	21(29.6)	50(70.4)	1.09(0.82–1.44)	0.566
30-	23(31.9)	49(68.1)	1.05(0.79–1.40)	0.741
Smoking years				
< 10	7(58.3%)	5(41.7%)	1.00	–
10-	17(34.0%)	33(66.0%)	1.58(0.79–3.19)	0.197
≥ 30	40(29.0%)	98(71.0%)	1.70(0.87–3.36)	0.123
Drinking				
Never	19(27.9%)	49(72.1%)	1.00	–
Once	16(35.6%)	29(64.4%)	0.89(0.69–1.16)	0.405
Occasionally	13(30.2%)	30(69.8%)	0.97(0.76–1.24)	0.797
Frequently	32(37.2%)	54(62.8%)	0.87(0.70–1.09)	0.220
Drinking index^d^				
0	32(32.3)	67(67.7)	1	–
1-	10(28.6)	25(71.4)	1.05(0.82–1.36)	0.672
50-	15(30.6)	34(69.4)	1.02(0.81–1.29)	0.832
100-	19(39.6)	29(60.4)	0.89(0.68–1.16)	0.404
Days of every week of intake of fruits				
< 1	7(18.4%)	31(81.6%)	1.00	–
1–3	27(35.5%)	49(64.5%)	0.79(0.63–0.99)	0.041
≥ 4	34(35.8%)	61(64.2%)	0.79(0.64–0.97)	0.028
Days of every week of physical exercise				
< 1	2(40.0%)	3(60.0%)	1.00	–
1-	11(28.9%)	27(71.1%)	1.18(0.56–2.49)	0.656
7	60(33.3%)	120(66.7%)	1.11(0.54–2.29)	0.775
Length of sleeping (hour)				
< 6	24(36.9%)	41(63.1%)	1.00	–
6-	34(29.8%)	80(70.2%)	1.11(0.89–1.39)	0.345
≥ 8	22(34.9%)	41(65.1%)	1.03(0.80–1.34)	0.813
History of BCG vaccination				
No	72(33.2%)	145(66.8%)	1.00	–
Yes	8(32.0%)	17(68.0%)	1.02(0.77–1.35)	0.904
The family history of tuberculosis				
No	78(33.5%)	155(66.5%)	1.00	–
Yes	2(22.2%)	7(77.8%)	1.17(0.82–1.68)	0.396
Exposure history of tuberculosis				
No	77(33.8%)	151(66.2%)	1.00	–
Yes	3(21.4%)	11(78.6%)	1.19(0.89–1.58)	0.246

*QFT* QuantiFERON-TB Gold In-Tube, *CI* Confidence Interval

^a^ Sum may not equal the total because of missing data

^b^Living space: average per-capita living space (m^2^)

^c^Smoking index(pack-years): average number of packs of cigarettes smoked per day multiplied by the number of smoking years

^d^Drinking index(50 g·year):grams of alcohol consumed daily multiplied by years of drinking

Job category was included in multivariate log-binomial regression analyses because it was related to LTBI borderline significantly. Table 3 showed the results from the multivariate log-binomial regression model. The poor workplace ventilation (APR: 1.26; 95% CI: 1.03–1.52) was found to be independent risk factor and ≥ 4 days of every week of intake of fruit (APR: 0.81; 95% CI: 0.65–0.99) was found to be independent protective factor associated with a positive QFT result. Job category was not associated with a positive QFT result.

**Table 3:** Multivariate log-binomial regression analysis of factors associated with QFT positivity among study participants (n = 242)

Variable	Adjust prevalence ratio	95% CI	p-value	p-trend
Job category				
Transport and helping	1.00	–		
Mining	1.49	0.80–2.76	0.207	
Tunneling	1.68	0.91–3.10	0.095	
Both tunneling and mining	1.70	0.92–3.13	0.090	
Workplace ventilation				
Well	1.00	–		
Poor	1.26	1.03–1.52	0.022	
Days of every week of intake of fruits				
< 1	1.00	–	–	0.033
1–3	0.82	0.66–1.03	0.089	
≥ 4	0.81	0.65–0.99	0.049	

*QFT* QuantiFERON-TB Gold In-Tube, *CI* Confidence Interval

## Discussion

In this study, we found that the prevalence of LTBI among CWP patients in China was 66.4% based on the QFT. This was considerably higher than that reported in a community-based study conducted in rural China (13%–20%) [13]. To date, besides a study that investigated the prevalence of LTBI among aged underground hard coal miners using IGRAs in Germany and reported positive rates of 46.6% (QFT) and 61.0% (T-SPOT) [11], no studies have reported the prevalence of LTBI among CWP patients in the rest of the world. As for occupational studies, a few reports employed the IGRA in healthcare workers and detected positive rates of 9.9% (QFT) in Japan [14], 10.6% (QFT) in Malaysia [15], 25% (QFT) in Italy [16], 40.8% (QFT) in Russia [17], 46% (QFT) in Georgia [18], 76.7% (QFT) and 65.7% (A.TB, a *Mycobacterium tuberculosis*-specific cell-mediated immune response detection kit) in China [8], and 46.0% (QFT) among village doctors in China [19]. The prevalence of LTBI in healthcare workers therefore varied greatly between countries, with a much higher LTBI rate in China than in any of the other countries.

In our study, 29.8% of CWP patients had a history of previous TB. This finding was in agreement with a previous study among 1107 patients in a coal mine industry group [20], which showed the total rate of pneumoconiosis complicated with tuberculosis was 30.5%.The prevalence (66.4%) of LTBI among CWP patients in our study was higher than among village doctors (46.0%) [19] but lower than that among healthcare workers (76.7%) in China [8]. This suggested that those exposed to TB via their occupation have a higher risk of LTBI in China. In our study, poor ventilation condition was positive associated with QFT positivity. These findings were in agreement with previous active TB studies [21, 22] that demonstrated that poor ventilation condition was vital risk factor for CWP with active TB. It is therefore clear that careful monitoring and dust control measures need to be improved in workplaces to ensure the safety of workers. Furthermore, physical examination and QFT tests should be performed regularly to facilitate the early detection of LTBI with CWP. Active treatment and better management of patients are required to reduce the risk of LTBI with CWP. We also found regular intake of fruit (≥4 days of every week) was associated with LTBI among CWP. Few studies have reported whether intake of fruits is significantly associated with LTBI. A case–control study in a community that investigated relation between specific dietary and susceptibility of tuberculosis, concluded that an inadequate intake of fruits and vegetables was associated with an increased risk of new tuberculosis infection [23]. However, large prospective and interventional studies are needed to confirm the effect of fruits.

To date, most community-based studies of LTBI have shown that a positive QFT result is associated with age. This has been shown in studies in rural China [13] and among healthcare workers [14, 24, 25]. However, in our study, this correlation was not detected among CWP patients. This may be explained by the fact that our study population predominantly comprised older participants who had survived CWP and who were all over 40 years of age (median age: 67 years). Studies have demonstrated a high risk of *M. tuberculosis* infection among individuals in close contact with TB cases [13, 22, 26]. However, in the present study, the positivity of QFT was not affected by a previous history of close contact with TB patients, and this was in agreement with the previous findings from some studies of healthcare workers [16, 25, 27]. This could be explained by the fact that some of the CWP patients in our study may have been unknowingly exposed to TB either within their working environment or in the community. Our study did not confirm the potential protective effect of BCG vaccination in CWP patients. This finding was consistent with the results of studies carried out in populations involved in other occupations [27, 28]. This finding may be explained by the fact that China has a high TB burden and a high coverage of BCG vaccination. However, BCG vaccination has been included in the national immunization program in China since 1978, so there would be poor vaccination coverage in participants born before that time possibly explaining the lack of impact of BCG vaccination among our study population. There is evidence showing that smoking is significantly associated with silicosis [29, 30] and with TB [22, 31, 32]; however, few studies have verified whether smoking is significantly associated with LTBI among CWP patients. In our study, smoking was not associated with LTBI.

### Limitations

There are several limitations of our study that should be addressed. Firstly, the sample size was limited. Secondly, participants were surviving CWP patients and the majority was over 60 years of age. Therefore, our study population is not representative of the general CWP population in China. Thirdly, the majority of participants had retired so it was difficult to verify their potential occupational contact with TB cases, leading to possible recall bias.

## Conclusions

This study showed that the high prevalence of LTBI among CWP patients in China. We found that poor workplace ventilation could be a risk factor and intake of fruits regularly (≥4 days of every week) found to be a protective factor. In response, specific measures need to be implemented including the proactive reduction of workers’ exposure to coal dust by improving ventilation conditions and regular screening of those at high risk of CWP to ensure the early detection of LTBI. Large studied should be studied for the effect intake of fruits regularly on the risk of LTBI.

## Additional file

Additional file 1: The results of QFT among 244 CWP patients. (XLSX 24 kb)

## Abbreviations

APR: Adjusted prevalence ratio
BCG: Bacillus Calmette – Guerin
BMI: Body mass index
CWP: Coal workers’ pneumoconiosis
ELISA: Enzyme-linked immunosorbent assay
IFN-γ: Interferon-γ
IGRAs: IFN-γ release assays
IQR: Interquartile range
LTBI: Latent tuberculosis infection
OD: Optical density
OR: Odds ratio; CI: confidence interval
PR: Prevalence ratio
QFT: QuantiFERON-TB Gold In-Tube
TB: Tuberculosis
TST: Tuberculin skin test
